# Human decidua basalis mesenchymal stem/stromal cells enhance anticancer properties of human natural killer cells, *in vitro*

**DOI:** 10.3389/fcell.2024.1435484

**Published:** 2024-10-30

**Authors:** Abdulaziz Almutairi, Najlaa A. Alshehri, Abdullah Al Subayyil, Eman Bahattab, Manal Alshabibi, Fawaz Abomaray, Yasser S. Basmaeil, Tanvir Khatlani

**Affiliations:** ^1^ College of Applied Medical Sciences, King Saud Bin Abdulaziz University for Health Sciences, Riyadh, Saudi Arabia; ^2^ Stem Cells and Regenerative Medicine Unit, Blood and Cancer Research Department, King Abdullah International Medical Research Center (KAIMRC), King Saud Bin Abdul Aziz University for Medical Sciences (KSAU-HS), King Abdulaziz Medical City (KAMC), Ministry of National Guard Health Affairs (MNGHA), Riyadh, Saudi Arabia; ^3^ School of Education, University of Alabama at Birmingham, Birmingham, AL, United States; ^4^ National Center for Stem Cell Technology, Life Sciences and Environment Research Institute, King Abdulaziz City for Science and Technology, Riyadh, Saudi Arabia; ^5^ Department of Clinical Science, Intervention and Technology, Division of Obstetrics and Gynecology, Karolinska Institutet, Stockholm, Sweden; ^6^ Center for Hematology and Regenerative Medicine, Karolinska Institutet, Stockholm, Sweden

**Keywords:** *decidua basalis* mesenchymal stem/multipotent stromal cells, NK cells, cytolytic activity, NK cell proliferation, cancer, inflammatory molecules, MCF-7, inflammatory molecules

## Abstract

**Introduction:**

Mesenchymal stem cells/stromal cells from the *Decidua Basalis* of the human placenta (DBMSCs) express wide range of effector molecules that modulate the functions of their target cells. These properties make them potential candidate for use in cellular therapy. In this study, we have investigated the consequences of interaction between DBMSCs and natural killer (NK) cells for both cell types.

**Methods:**

DBMSCs were cultured with IL-2-activated and resting non-activated NK cells isolated from healthy human peripheral blood and various functional assays were performed including, NK cell proliferation and cytolytic activities. Flow cytometry and microscopic studies were performed to examine the expression of NK cell receptors that mediate these cytolytic activities against DBMSCs. Moreover, the mechanism underlying these effects was also investigated.

**Results:**

Our findings revealed that, co-culture of DBMSCs and NK cells resulted in inhibition of proliferation of resting NK cells, while proliferation of IL-2 activated NK cells was increased. Contrarily, treatment of DBMSC’s with comparatively high numbers of IL-2 activated NK cells, resulted in their lysis, whereas treatment with low numbers resulted in reduction in their proliferation. Cytolytic activity of NK cells against DBMSCs was mediated by several activating NK cell receptors. In spite of the expression of HLA class I molecules by DBMSCs, they were still lysed by NK cells, excluding their involvement in cytolytic activity. In addition, preconditioning NK cells by DBMSCs, enhanced their ability to suppress tumor cell proliferation and in severe cases resulted in their partial lysis. Lysis and decrease of tumor cell proliferation is associated with increased expression of important molecules involved in anticancer activities.

**Discussion:**

We conclude that DBMSCs exhibit dualfunctions on NK cells that enhance their anticancer therapeutic potential.

## Introduction

We have previously reported isolation and characterization of mesenchymal stem cells/stromal cells (MSCs) from the maternal *decidua basalis* tissue (DBMSCs) of human full-term placentae (Abomaray et al., 2016). DBMSCs have therapeutic potential to treat cardiovascular diseases, such as atherosclerosis through a mechanism that involves protecting endothelial cells from injuries caused by oxidative stress mediators including hydrogen peroxide and monocytes (Alshabibi et al., 2017; Alshabibi et al., 2018). In addition, DBMSCs have a potential to treat cancers by enhancing differentiation of M1 macrophages and inflammatory immune cells with anticancer activity (Abumaree et al., 2019a). These properties of DBMSCs make them potential therapeutic agents against cancer.

The interaction between NK cells (natural killer cells) and MSCs from the maternal side; *decidua parietalis* (DPMSCs) and from the fetal part, the *chorionic villi* (pMSCs) of human term placenta, were recently reported (Abomaray et al., 2016). These interactions resulted in lysis of both DPMSCs and pMSCs (Abumaree et al., 2018; Abumaree et al., 2019a). Importantly, both cell types enhanced the anticancer activities of NK cells, *in vitro* (Abumaree et al., 2018; Abumaree et al., 2019a). However, the NK cell modulatory effects of DBMSCs and *vice versa*, are currently unknown.

NK cells are immune cells with cytotoxic or cytolytic activities against cancer cells (Brandstadter and Yang, 2011). These activities are mediated by several cell-surface activating molecules, such as NKp30, NKp44, NKp46, DNAM-1 and NKG2D, and inhibitory receptors such as, killer immunoglobulin-like receptors “KIRs” and CD94/NKG2A, that mediate their functions on target cells (Moretta et al., 1996; Shibuya et al., 1996; Braud et al., 1998; Bauer et al., 1999; Wu et al., 1999; Cosman et al., 2001; Pende et al., 2002; Bottino et al., 2003; Moretta and Moretta, 2004). In addition, anti-cancer activities of NK cells are also mediated by different factors that include cytokines; their corresponding receptors, and Toll-like receptors (TLRs) (Moretta et al., 2001; Sivori et al., 2004; Adib-Conquy et al., 2014).

In this study, we examined the consequences of DBMSCs interaction with human NK cells and found that DBMSCs were susceptible to lysis when cultured together with NK cells. In addition co-culturing of NK cells with DBMSCs increased expression of various molecules on NK Cells, which are involved in anticancer activities. Based on the results, we conclude that DBMSCs have the potential to enhance NK cells’ anticancer therapeutic activity.

## Material and methods

### Collection of human placentae and human peripheral blood

The Institutional Review Board (IRB) at King Abdullah International Medical Research Centre (KAIMRC), Saudi Arabia approved this study. Placentae from uncomplicated human pregnancies (38–40 weeks of gestation) and peripheral blood samples from healthy human adult subjects were collected after signing an informed consent form. The placentae were used immediately after delivery. Blood samples were processed to isolate NK cells.

### Isolation and culture of DBMSCs

DBMSCs were isolated from the *decidua basalis* of full-term placentae using an established protocol of our laboratory. In brief, the cells were cultured in DMEM culture medium containing; Dulbecco’s modified Eagle’s medium nutrient mixture F-12 (DMEM-F12), 10% Mesenchymal Stem Cell certified fetal bovine serum (MSC FBS) (Life Technologies, Grand Island, United States), and antibiotics (100 μg/mL streptomycin and 100U/mL penicillin). The cells were incubated at 37°C in a humidified atmosphere containing 5% CO_2_ and 95% air (in a cell culture incubator). DBMSCs (passage 3) of twenty placentae were used in subsequent experiments.

### Isolation of human NK cells

NK cells were isolated from human peripheral blood (PBMNCs) of 10 healthy subjects as previously described (Abumaree et al., 2018; Abumaree et al., 2019a). Trypan blue was used to determine their viability, and purity was assessed using anti-CD56 monoclonal antibody (R and D Systems, Abingdon, United Kingdom) in a flow cytometer (Abumaree et al., 2018; Abumaree et al., 2019b). The viability and purity of NK cells used in this study were more than 95%. The isolated NK cells were used either immediately, or after 72 h of activation with 100U/mL IL-2 (R and D Systems, Abingdon, United Kingdom) in RPMI 1640 medium containing 10% FBS, 2 mM L-glutamine, and the antibiotics indicated above (NK culture medium).

### NK cell proliferation assay

NK cell proliferation was evaluated by MTS assay [3-(4, 5-dimethylthiazol-2-yl)-5-(3-carboxymethoxyphenyl)-2-(4-sulfophenyl)-2H-tetrazolium, inner salt] (cat #G5421, Promega, WI, United States)] as previously described (Abumaree et al., 2018; Abumaree et al., 2019b). Briefly, resting (non-activated) NK cells (NK cells induced to proliferate by 100U/mL IL-2) or activated NK cells (NK cells pre-cultured with 100U/mL IL-2 for 72 h) were cultured with or without DBMSCs at different NK to DBMSC ratios, ranging from 1:1 to 15:1, 25:1, 50:1, and 100:1. All NK cell/DBMSC cultures were carried out using the NK culture medium, as described above. Prior to the addition of DBMSCs to NK cell proliferation experiments, DBMSCs proliferation was inhibited by treating them with Mitomycin C. After 3 days of culture, NK cell proliferation was determined as described earlier (Abumaree et al., 2018; Abumaree et al., 2019b). The results from triplicate samples were presented as the means ± standard errors. Different time points were evaluated to assess NK proliferation. Experiments were performed in triplicates and repeated 10 times using 10 individual preparations of both NK cells and the DBMSCs.

### NK cell cytolytic assays

To determine the cytolytic potential of NK cells against DBMSCs, IL-2-non-activated NK cells and activated NK cells (NK cells pre-cultured with 100U/mL IL-2 for 72 h) were cultured with DBMSCs at different ratios of NK to DBMSCs, i.e., 1:1, 15:1, 25:1, 50:1 and 100:1 NK: DBMSC. NK cell activation was performed as previously reported (Abumaree et al., 2018; Abumaree et al., 2019b). Briefly, DBMSCs were seeded in a 6-well plate in complete DBMSC culture medium at 37°C for 24 h. Adherent DBMSCs were treated with IL-2 (activated and non-activated) NK cells at different ratios of NK: DBMSCs and incubated at 37°C. For blocking assays, NK cells were pre-incubated with a monoclonal antibody specific to different NK cell receptors (activating and inhibitory) (Table 1) at 10 μg/mL (final concentration) (Abumaree et al., 2018; Abumaree et al., 2019b). After incubation for 30 min at 4°C, the cells were washed and assayed for cytolytic experiments at 100:1 (NK: DBMSC) ratio. Flow cytometry was used to identify DBMSC surface expression of ligands (Table 2) recognized by NK cell activating or inhibiting receptors (Abumaree et al., 2018; Abumaree et al., 2019b). NK cells or DBMSCs cultured alone were used as controls. Cell lysis was assessed using light microscopy and photomicrographs were recorded. Lysis of cells was determined by calculating the percentage of the remaining intact adherent cells, per well (Abumaree et al., 2018; Abumaree et al., 2019b). Experiments were repeated three times using fresh preparations of NK cells and DBMSCs as indicated above.

**TABLE 1:** Monoclonal antibodies used in the blocking experiments.

Monoclonal antibody target protein	Isotype	Clone	Catalogue number
NKp30	IgG2A	210,847	MAB 1849
NKp44	IgG2a	253,415	MAB22491
NKp46	IgG2b	195,314	MAB 1850
CD69	IgG2a	298,633	MAB23591
CD226 (DNAM-1)	IgG1	102,511	MAB666
NKG2D (CD314)	IgG1	149,810	MAB139
CD94	IgG1	131,412	MAB1058

**TABLE 2:** Antibodies of the protein targets used in this study.

HLA-markers	NK cell markers	NK cell activating receptors	NK cell inhibitory receptors	NK cell receptor ligands	Immune proteins
HLA-ABC	CD56	CD69	CD94/NKG2A	PVR (CD155)	IL-12Rβ1
		CD226		Nectin-2 (CD112)	IL18Rα
		NKp30		ULBP-1	IL-18Rβ
		NKp44		ULBP-2	IFN-ɣR1
		NKp46		ULBP-3	IFN-ɣR2
		NKpG2D (CD314)		MICA	TLR3
				MICB	TLR7
				HLA-E	TLR9
					TNF-α

### Cytolysis of tumor cells by NK cells

To examine whether NK cell cytolytic activity was affected following their co-culture with DBMSCs for 50 h, IL-2-activated NK cells cultured alone or co-cultured with DBMSCs at 15:1, 25:1, 50:1 and 100:1 pertaining to NK: DBMSC ratios in the NK/DBMSC cytolytic experiment, were harvested and purified followed by assessing their viability and purity. CD56^+^ NK cells with viability and purity of more than 95% were used against MCF-7 cells (breast cancer cells, ATCC, Manassas, VA, United States) at a ratio of 10:1 NK to MCF-7 cells, as previously described (Abumaree et al., 2018; Abumaree et al., 2019b). Cells were cultured and incubated in NK culture medium, to evaluate their lysis. NK cells or MCF-7 cells cultured alone were considered as controls. Different ratios of NK: MCF-7 cells were also evaluated (data not shown), but a ratio of 10:1 NK: MCF-7 cells was chosen because majority of MCF-7 cells were lysed at that ratio (Abumaree et al., 2018; Abumaree et al., 2019b). Triplicate experiments were performed each time and repeated 10 times using NK cells isolated from 10 independent DBMSC/NK cytolytic assays and MC7 breast cancer cells.

### NK cell Cytolytic Activity against DBMSCs and Tumor cells using the xCelligence Real-Time Functional Assay

To study the cytolytic activities of NK cells against DBMSCs or MCF-7 cells, a real time cell analyzer (xCelligence RTCA-DP, Roche Diagnostics, Mannheim, Germany) was used as previously published (Rocca et al., 2016; Abumaree et al., 2017a; Abumaree et al., 2018; Abumaree et al., 2019b). For the NK cell/DBMSC cytolytic experiment, IL-2-non-activated or -activated NK cells were used against DBMSCs at different ratios (15:1, 25:1, 50:1 and 100:1 NK: DBMSC ratios). For the NK cell/MCF-7 cytolytic experiment, IL-2-activated NK cells cultured with DBMSCs in the NK cell/DBMSC cytolytic experiment (see above), were used against MCF-7 cells. After incubation of NK cells with DBMSCs, the cells were isolated and their viability and purity assessed before using them against MCF-7 cells at a ratio of 10:1 NK: MCF-7 cells. Sixteen-well E-culture plates (cat# 05469813001, Roche Diagnostics) were used in both cytolytic experiments, and background impedance was measured (Rocca et al., 2016; Abumaree et al., 2017b; Abumaree et al., 2019b). For each experiment, 2 × 10^4^ DBMSCs or MCF-7 cells, with or without NK cells at the indicated ratios, were seeded in an NK culture medium in quadruplicate wells and left to equilibrate before data recording. After incubation at 37°C, the cell index of the DBMSC/NK cell or MCF-7/NK cell cultures was monitored in real time. The xCelligence software (version 1.2.1) was used to analyze cell proliferation data (cell index) and expressed as the mean ± SD. The rate of cell growth was determined by calculating the linear regression of the slopes between two given time points. Five experiments were carried out using DBMSCs (P3) and NK cells (n = 5 independent preparations each, with quadruplicate samples).

### NK cell expression of activating and inhibitory receptors and immune proteins

To examine DBMSC effects on NK cell expression of activating and inhibitory receptors, as well as immune proteins (Table 2), NK cells were harvested from the cytolytic assay and purified using the NK Cell Isolation Kit and the magnetic cell separator as described above. Flow cytometry was used to characterize NK cells.

### Flow cytometry

Cells (1 × 10^5^) were stained with the antibodies listed in Table 2, for 30 min, and then, flow cytometry for cell surface and intracellular proteins was performed using a BD FACS CANTO II (Becton Dickinson) flow cytometer as previously described (Alshabibi et al., 2017). Negative controls were cells stained with FITC or PE-labeled mouse IgG isotype antibody.

### Statistical analysis

GraphPad Prism 5 was used to analyze data. Data are shown in bar and line graphs as means ± standard error (SE) from three independent experiments. Non-parametric tests, including the Mann-Whitney U test for comparing two groups and the Kruskal–Wallis test for comparing three or more groups, were used. Categorical data were compared using chi-square tests, while ANOVA was applied for comparisons involving more than two groups. A *p*-value of ≤0.05 was considered statistically significant”.

## Results

### DBMSCs inhibit NK cell proliferation

**FIGURE 1:**
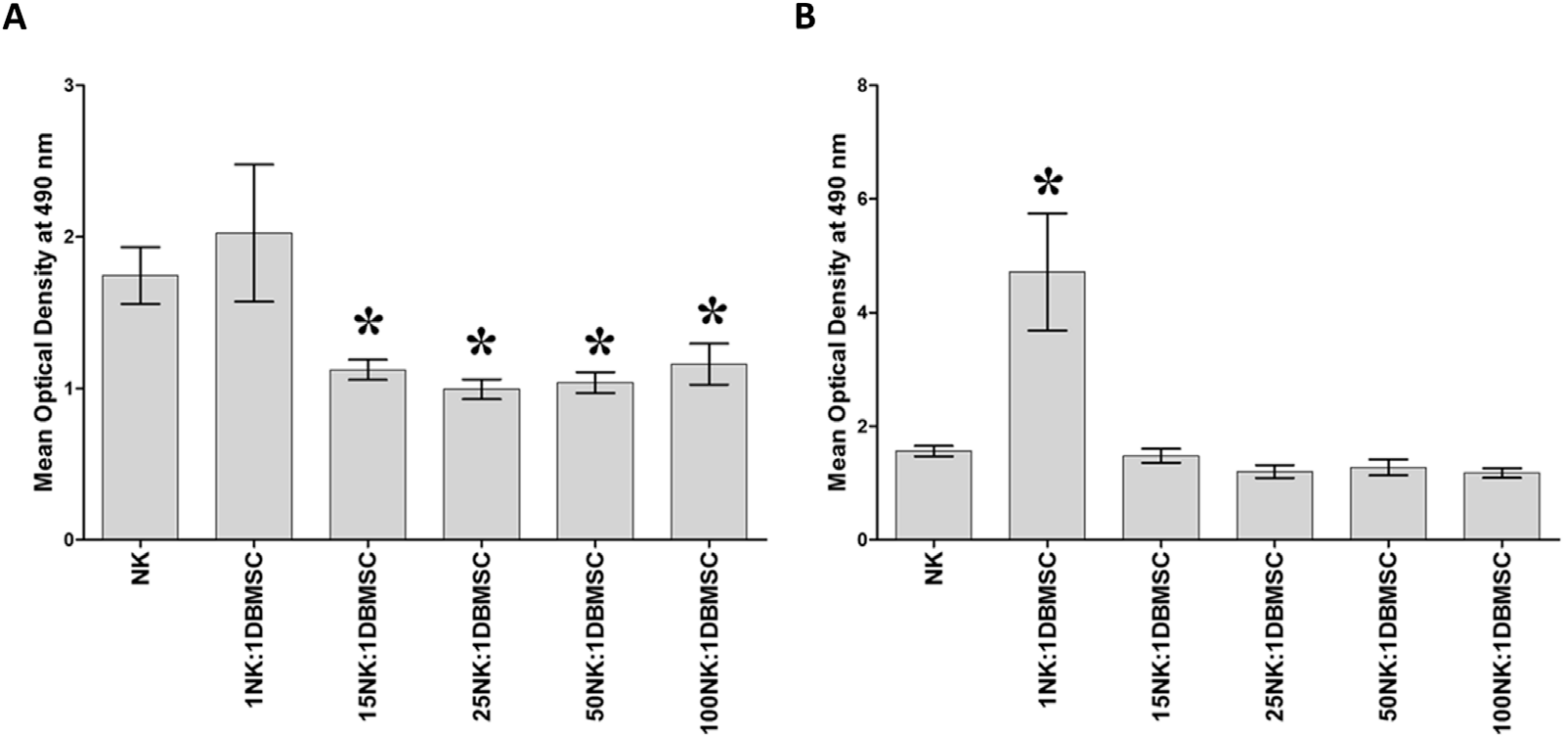
The effect of DBMSCs on NK cell proliferation. **(A)** The proliferation of resting non-activated cells (NK cells were induced to proliferate by 100 U/mL IL-2) was significantly decreased in presence of DBMSCs relative to untreated resting non-activated NK cells, at all ratios shown except for the 1NK:1DBMSC ratio. **(B)** IL-2-activated NK cell proliferation significantly increased at the 1NK:1DBMSC ratio in the presence of DBMSCs relative to untreated IL-2-activated NK cells. NK cells were initially activated by 100 U/mL IL-2 for 72 h. Results of 10 representative experiments are shown, in which the proliferation of resting non-activated or IL-2-activated NK cells cultured for 72 h, with or without Mitomycin C-treated DBMSCs, was measured at the NK: DBMSC ratios of 1:1, 15:1, 25:1, 50:1 and 100:1, using the MTS proliferation method. Experiments were performed in triplicates using NK cells and DBMSCs prepared from the peripheral blood of 10 healthy subjects and 10 normal human full-term placentae, respectively. Bars represent standard errors, *P< 0.05.

DBMSCs at passage 3 from the decidua basalis of human full-term placentae were used in this study. To determine the effect of DBMSCs on NK cell proliferation, we cultured DBMSCs with resting non-activated NK cells (NK cells induced to proliferate by 100 U/mL IL-2) or activated NK cells (NK cells pre-cultured with 100 U/mL IL-2 for 72 h). After 72 h of co-culture with DBMSCs, the proliferation of resting non-activated NK cells was significantly inhibited at NK:DBMSC ratios of 15:1 to 100:1, relative to NK cells cultured alone (P< 0.05, Figure 1A). In contrast, at a ratio of 1 to 1 (NK: DBMSC), DBMSCs increased the proliferation of activated NK cells (P< 0.05), but showed no effects on NK cell proliferation at ratios ranging from 15:1 to 100:1 NK:DBMSCs, relative to NK cells cultured alone (Figure 1B).

### DBMSC express ligands that bind to NK cell receptors

Activating receptors mediate NK cell cytolytic activity, which is arbitrated by their binding to their corresponding ligands. They include poliovirus receptor (PVR) and Nectin-2 for DNAM-1, and MICA/B (MHC class I chain-related gene A and B) and ULBPs (UL16 binding proteins) for NKG2D (Moretta et al., 1996; Shibuya et al., 1996; Bauer et al., 1999; Wu et al., 1999; Cosman et al., 2001; Pende et al., 2002; Bottino et al., 2003; Moretta and Moretta, 2004). Binding of NK KIRs and CD94/NKG2A receptors to HLA-Class I (Human Leukocyte Antigen class I) and to HLA-E (non-classical MHC class 1), respectively, inhibits NK cytolytic activity against the cells expressing these molecules (Moretta et al., 1996; Braud et al., 1998). Conversely, target cells lacking HLA molecules, this ligand-receptor binding results in the activation of NK cells, which in turn induces killing or lysis of the target cells (Ljunggren and Kärre, 1990). Therefore, the potential interaction between NK cells and DBMSCs was examined by evaluating the expression of ligands by DBMSCs that are known to bind to different NK cell (activating and inhibiting) receptors. The percentage of DBMSCs that express these ligands are shown in Table 3. The NK activating receptor ligand that was most highly expressed by DBMSCs was PVR (also known as CD155), which binds to DNAM (22.00% ± 9.07%). Ligands for other activating receptors were expressed at very low levels by DBMSCs, ranging from 0.76% ± 0.38%–4.63% ± 2.19% of expressing cells. By contrast, 22.00% ± 9.07% and 97.00% ± 3.00% of DBMSCs expressed the NK cell inhibitory receptor ligands for HLA-E and HLA-ABC, respectively.

**TABLE 3:** Percentage of DBMSCs expressing surface ligands that bind to NK-activating and NK-inhibiting receptors.

DNAM-1 activating receptor ligands		NKG2D activating receptor ligands					CD94/NKG2 inhibiting receptor ligand	
PVR (CD155)	Nectin-2 (CD112)	ULBP-1	ULBP-2	ULBP-3	MICA	MICB	HLA-E	HLA-ABC
22.00% ± 9.07%	0.76% ± 0.38%	2.067% ± 0.533%	4.63% ± 2.19%	1.93% ± 0.83%	2.66% ± 2.18%	0.96% ± 0.966%	22.00% ± 9.07%	97.00% ± 3.00%

### DBMSC lysis by NK cells

**FIGURE 2:**
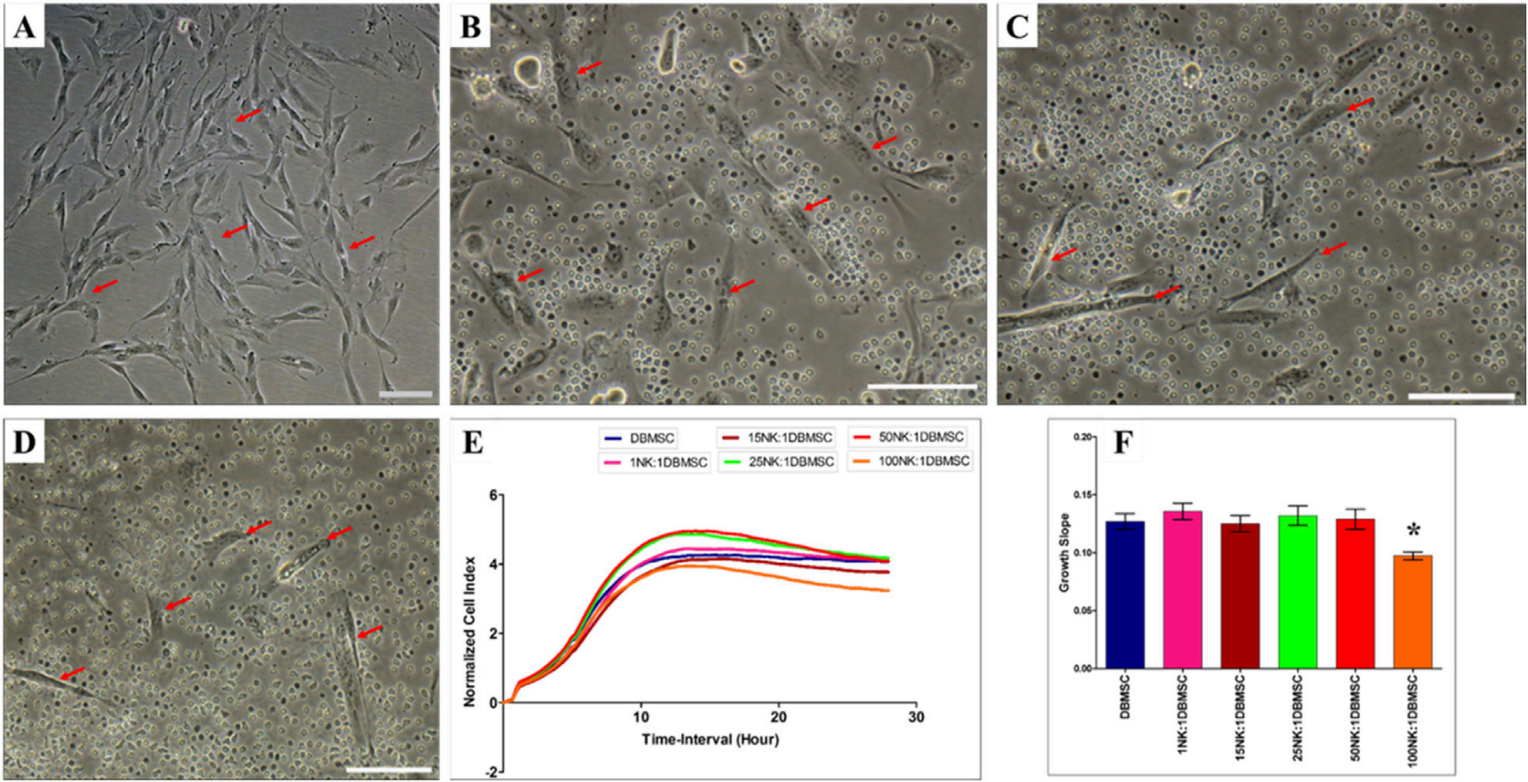
NK cell interaction with DBMSCs. NK cell interaction with DBMSCs (evaluated by culturing IL-2 non-activated NK cells with DBMSCs at different NK: DBMSC ratios for 28 h) and NK cell cytolytic activity against DBMSCs were evaluated using microscopic examination and the xCelligence real-time cell system. Representative phase contrast microscopic images show that untreated DBMSCs (control) have a spindle-like morphology **(A)** Non-lysed and intact DBMSCs (arrow) cultured with IL-2 non-activated NK cells are shown at NK: DBMSC ratios of 25:1 **(B)**, 50:1 **(C)**, and 100:1 **(D)** The results of the xCelligence showing that after 28 h of culture, IL-2-non- activated NK cells did not lyse DBMSCs, as reflected by the cell index **(E)** and decreased DBMSC proliferation (growth slope) at a NK:DBMSC ratio of 100:1, relative to untreated DBMSCs (control), *P< 0.05, **(F)**. Experiments were performed in triplicates and repeated 10 times using NK cells and DBMSCs prepared from the peripheral blood of 10 healthy donors and 10 normal human full-term placentae, respectively. Scale bars: 50 µm.

**FIGURE 3:**
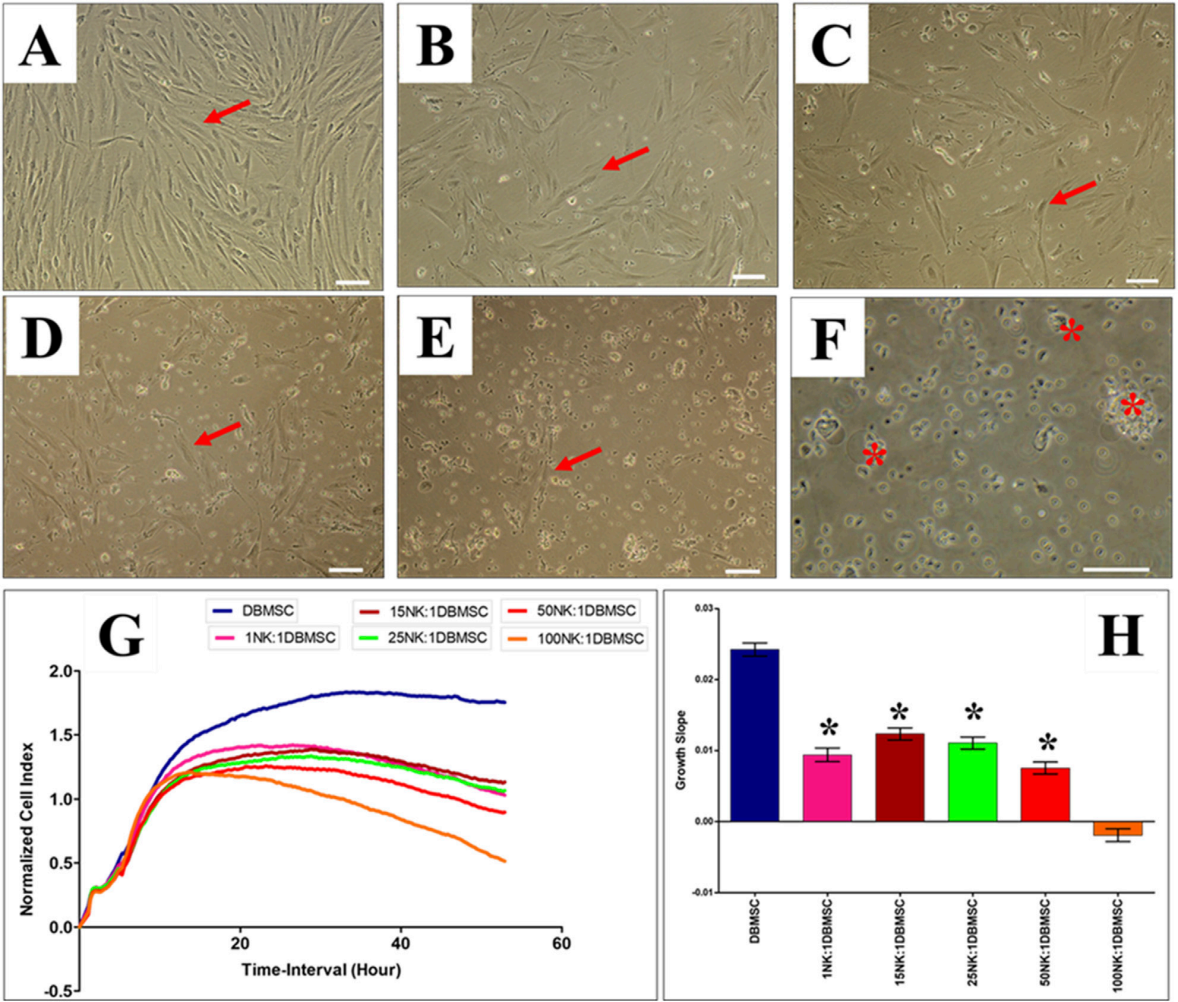
IL-2 activated NK cells lyse DBMSCs. The interaction of IL-2-activated NK cells with DBMSCs in culture was evaluated at different NK: DBMSC ratios and their cytolytic activity assessed within 50 h of co-culture by microscopic examination and using the xCelligence real time cell system. Representative phase contrast microscopic images showing untreated DBMSCs (control) with typical spindle-like morphology **(A)**. Non lysed DBMSCs (arrow) cultured with IL-2 activated NK at NK cell: DBMSC ratios of 1:1 **(B)**, 15:1 **(C)**, 25:1 **(D)**, 50:1 **(E)** and lysed DBMSC (arrow) cultured with IL-2 activated NK cells at a NK:DBMSC ratio of 100:1 **(F)**. The lysis of DBMSCs was determined by the lack of intact adherent DBMSCs and cell rupture, as determined by the presence of cellular debris in suspension. The results of the xCelligence show that after 50 h culture, IL-2-stimulated NK cells did not lyse DBMSCs at NK: DBMSC ratios ranging from 1:1 to 50:1, but DBMSCs were lysed at a NK:DBMSC ratio of 100:1, as reflected by a cell index of almost zero, indicating that DBMSCs were not intact (no adhesion and proliferation) **(G)** and by the growth slope **(H)**. At NK cell: DBMSC ratios ranging from 1:1 to 50:1, DBMSC proliferation significantly decreased relative to DBMSCs cultured alone, *P< 0.05 **(G, H)**. Experiments were performed in triplicates and repeated 10 times using NK cells and DBMSCs prepared from the peripheral blood of 10 healthy donors and 10 normal human full-term placentae, respectively. Scale bars: 50 µm.

Since DMBSCs express some ligands that bind to NK cell activating receptors, suggesting that DBMSCs are most likely to be lysed by NK cells. Accordingly, we investigated whether IL-2- activated and non-activated NK cells lyse DBMSCs. In agreement with our previous studies (Alshabibi et al., 2018; Abumaree et al., 2019b) and others (Rasmusson et al., 2003; Spaggiari et al., 2006), IL-2-non-activated NK cells did not lyse DBMSCs (Figures 2B–F) as compared to untreated control (Figure 2A). However, NK cells significantly reduced DBMSC proliferation at a 100:1 (NK: DBMSC) ratio (Figures 2E, F, P< 0.05) after 28 h. In contrast, following the culture of DBMSCs with IL-2-activated NK cells at a 100:1 (NK: DBMSC) ratio for 72 h, NK cells were able to completely lyse DBMSCs (as reflected by the absence of intact adherent DBMSCs, and the presence of ruptured cells and cellular debris in the culture suspension within 50 h) (Figure 3F). At the 50:1 ratio, NK cells partially lysed DBMSCs (Figure 3E). DBMSC lysis by NK cells was also confirmed by the xCelligence real time cell system. The cell index, which usually shows the adhesion and proliferation of cells, was below one, reflecting the lysis of cells (Figures 3G, H). However, at the 1:1, 15:1, and 25:1 ratios, DBMSCs were not lysed by the NK cells (Figures 3B–D, G, H) as compared to untreated control (Figure 3A), instead, their proliferation was significantly reduced (Figures 3G, H, P< 0.05).

### The NK cell receptors that contribute to DBMSC lysis

**FIGURE 4:**
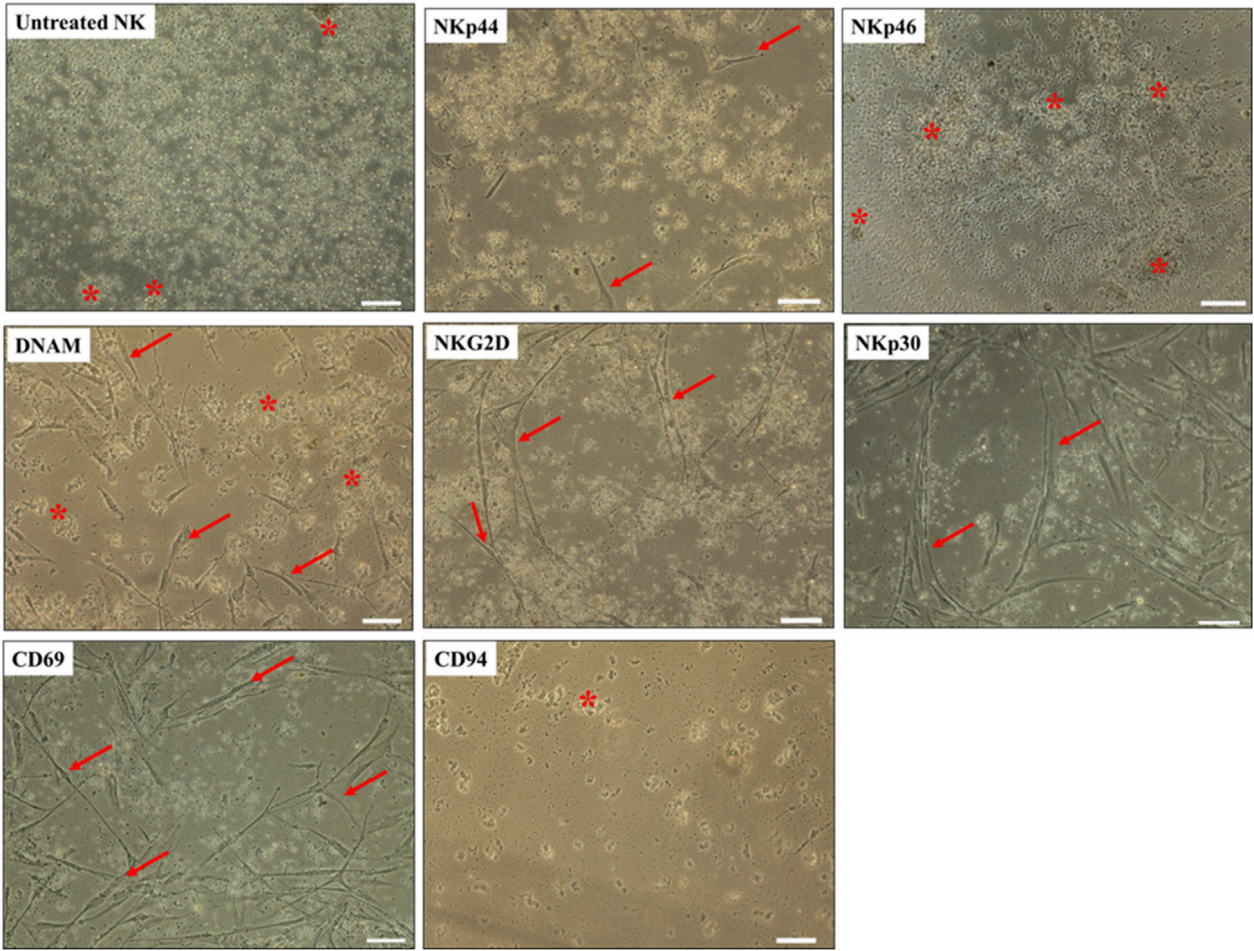
NK-cell activating and inhibiting receptors mediate DBMSC lysis. NK cells were pre-incubated with antibodies specific to the indicated NK-cell activating and inhibiting receptors. NK cells were then co-cultured with DBMSCs at a NK: DBMSC ratio of 100:1 in a cytolytic experiment. DBMSC lysis by NK cells was evaluated by microscopic examination within 24 h of culture. Representative phase contrast microscopic images show complete lysis of DBMSCs, as determined by the lack of intact adherent DBMSCs and cell rupture, indicated by the presence of cellular debris in suspension (stars indicate ruptured DBMSCs). DBMCs after treatment with untreated NK cells (NK cells were initially activated with 100 U/mL IL-2 for 72 h). Following pre-incubation with antibodies against NKp44, NKp46, and DNAM, a ∼50% inhibition of NK cell-mediated lysis of DBMSCs was recorded, with ∼50% of DBMSCs remaining adherent. Following pre-incubation with antibodies against NKG2D, CD69 and NKp30, 25% inhibition of NK cell lysis of DBMSCs occurred, with ∼75% of DBMSCs being adherent. Blocking CD94 receptor did not increase the cytolytic activity of NK cells. Experiments were performed in triplicates and repeated 10 times using NK cells and DBMSCs prepared from the peripheral blood of 10 healthy donors and 10 normal human full-term placentae. Scale bars: 50 µm.

To identify the receptors on NK cells that mediate lysis of DBMSCs, we used the monoclonal antibody-mediated blocking method in cytolytic experiments, as previously described (Abumaree et al., 2018; 2019b). In these experiments, NK cells were co-cultured with allogenic DBMSCs for 50 h. The antibody-mediated blocking of the receptors, NKp44 and NKp46, did not prevent the lysis of DBMSCs by NK cells (as reflected by the lack of adherent DBMSCs and cellular debris in suspension) (Figure 4). In contrast, the blocking of the DNAM and NKG2D receptors resulted in approximately 50% inhibition of DBMSC lysis by NK cells (∼50% of DBMSCs were adherent), while blocking of CD69 and CD30 receptors resulted in an approximately 25% inhibition of DBMSC lysis by NK cells (∼75% of DBMSCs were adherent) (Figure 4). Blocking of CD94/NKG2A (the HLA-E specific inhibitory receptor) did not affect NK cell cytolytic activity against DBMSCs (Figure 4).

### Functional activities of NK cells preconditioned with DBMSCs

**FIGURE 5:**
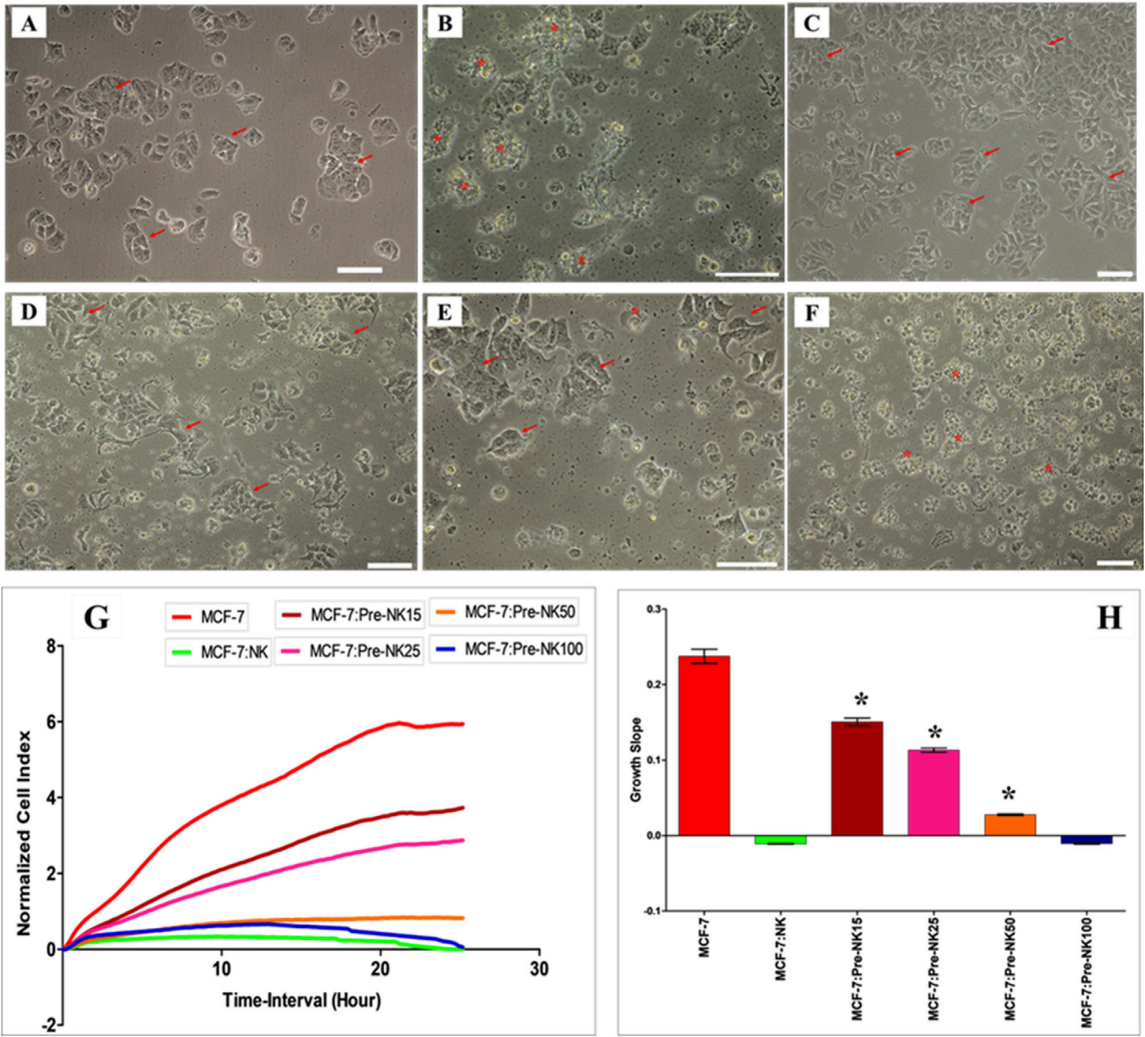
Interaction of activated NK cells with MCF-7 breast cancer cells. NK cells were initially activated with 100 U/mL IL-2 for 72 h and then cultured with DBMSCs at different NK: DBMSC ratios in a cytolytic experiment. Following 50 h of incubation with DBMSCs, NK cells (Pre-NK) were harvested, purified and added to MCF-7 cells at a ratio of 10:1 (NK: MCF-7). NK cytolytic activity against MCF-7 cells was then evaluated by microscopic examination and using the xCelligence real-time cell system. Representative phase contrast microscopic images showing **(A)** MCF-7 cells (arrows) cultured alone and **(B)** a complete lysis of MCF-7 cells (denoted by stars, and as determined by a lack of intact adherent MCF-7 and ruptured cells, indicated as cellular debris in suspension) by untreated NK cells within 24 h of culture, relative to MCF-7 controls. No lysis of MCF-7 cells was seen when activated NK cells were pre-cultured with DBMSCs at the NK: DBMSC ratios of **(C)** 15:1; **(D)** 25:1; and **(E)** 50:1. **(F)** At a ratio of 100:1, complete lysis of MCF-7 cells by activated NK cells pre-cultured with DBMSCs was observed. **(G, H)** The results of the xCelligence assay show that after 24 h of culture, MCF-7 were completely lysed by untreated NK cells and by NK cells pre-cultured with DBMSCs at a NK: DBMSC ratio of 100:1, as reflected by the cell index, which showed cell growth reduced to almost zero for MCF-7, indicating the loss of intact cells. MCF-7 proliferation was also significantly decreased by NK cells pre-cultured with DBMSCs at NK: DBMSC ratios ranging from 1:1 to 50:1. Experiments were performed in triplicates using the indicated NK: DBMSC ratios, using NK cells harvested from 10 independent NK/DBMSC cytolytic assays and MC7 breast cancer cells. Scale bars represent 50 µm.

To further study the possible modulatory effects of DBMSCs on NK cell cytolytic activity, NK cells were activated with IL-2 for 72 h and used in the NK cell/DBMSC cytolytic experiment at the ratios of 15:1, 25:1, 50:1 and 100:1 (NK: DBMSC). Following the co-culture of NK cells using a non-contact culture experiment, where a transwell 0·4 µm plate (Falcon, NJ, United States) was used. The NK cells were cultured in the outer chamber, and the DBMSCs were cultured in the inner chamber. After incubation for 50 h, the cells were harvested carefully from each side of the insert without any chance of cross contamination or mixing for subsequent experiments. NK cells were harvested, purified and co-cultured with MCF-7 breast cancer cells in an NK cell/MCF-7 cytolytic experiment at a ratio of 10:1 (NK: MCF-7). IL-2-treated NK cells cultured without DBMSCs served as a control. As shown in Figure 5, and compared to MCF-7 cells cultured alone, NK cells cultured with and without DBMSCs at a 100:1; NK: DBMSC ratio completely lysed MCF-7 cells (as reflected by a lack of intact adherent MCF-7 cells and cellular debris in suspension) within 24 h. This cytolytic activity of NK cells against MCF-7 cells was also confirmed using the xCelligence real time system. The cell index for MCF-7 cells co-cultured with NK cells pre-cultured with DBMSCs at a 100:1 ratio or cultured alone was zero, demonstrating a lack of intact MCF-7 cells (and thus cell lysis) (Figures 5G, H). However, NK cells pre-cultured with DBMSCs at the ratios of 15:1, 25:1 and 50:1, were unable to lyse MCF-7 cells (Figures 5C–E, G, H), but at these ratios, NK cells did significantly reduce MCF-7 cell growth (Figures 5G, H, *P< 0.05).

### Immune marker expression by NK cells Co-cultured with DBMSCs

**FIGURE 6:**
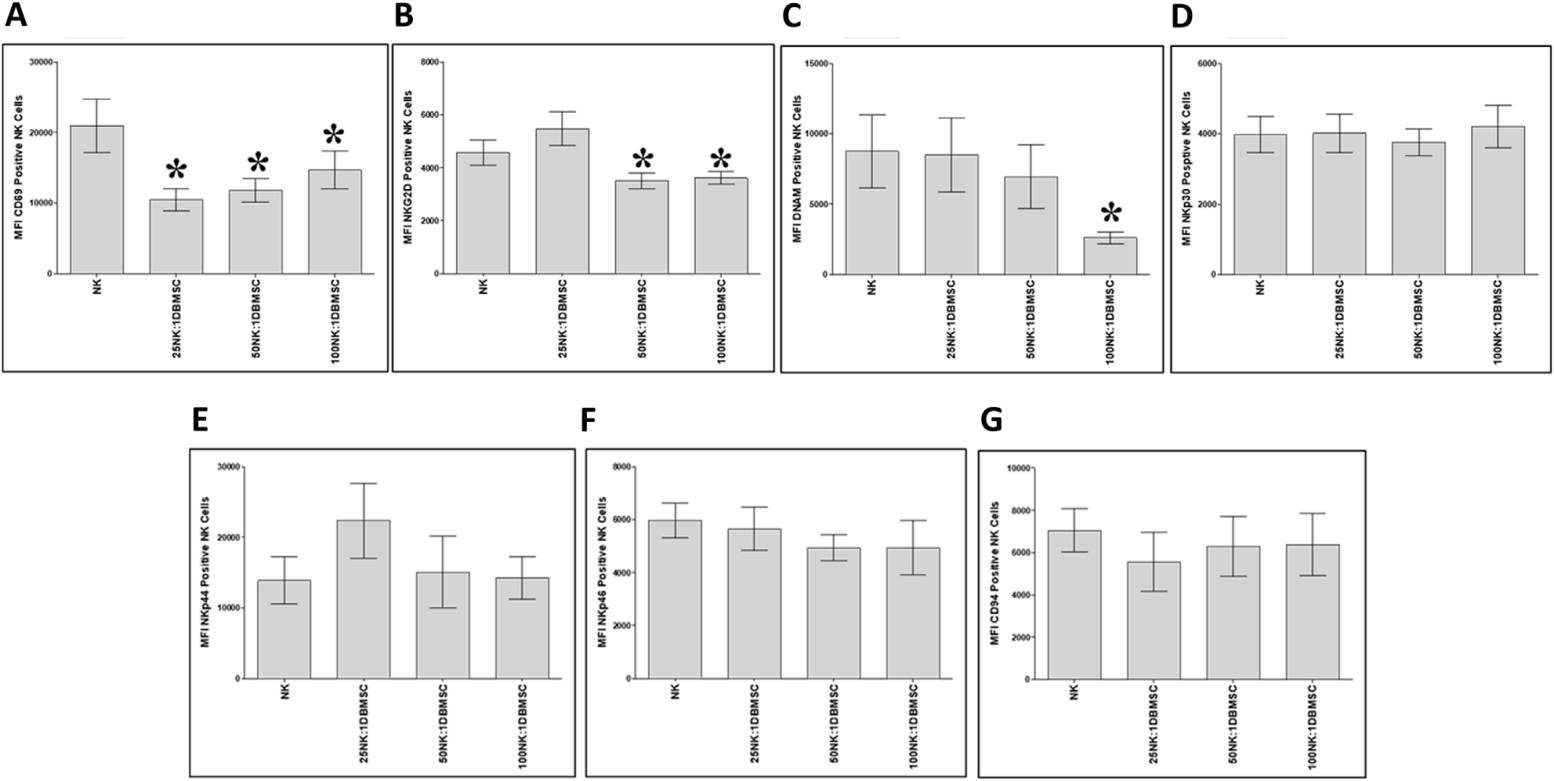
NK cell immune receptor expression following co-culture with DBMSCs. Using flow cytometry, the expression of activating and inhibiting receptors was evaluated in NK cells harvested from the NK/DBMSC cytolytic experiments. Compared with untreated NK cells, NK cells co-cultured with DBMSCs significantly decreased their expression of **(A)** CD69 at all NK:DBMSC ratios (25:1, 50:1 and 100:1), **(B)** NKG2D at the NK cell: DBMSC ratios of 50:1 and 100:1, and **(C)** DNAM at the NK: DBMSC ratio of 100:1. The co-culture of NK cells with DBMSCs had no significant effect on their expression of **(D)** NKp30, **(E)** NKp44, **(F)** NKp46 or **(G)** CD94 at any of the indicated NK: DBMSC ratios, P> 0.05. Experiments were performed in triplicates and repeated 10 times using 10 independent NK/DBMSC cytolytic experiments, where the NK cells and DBMSCs for each experiment were prepared from the peripheral blood of 10 healthy subjects and 10 normal human full-term placentae, respectively. Bars represent standard errors, *P< 0.05.

To examine whether the phenotype of IL-2-activated NK cells in the NK/DBMSC cytolytic experiment was modified after being co-cultured with DBMSCs at 25:1, 50:1 and 100:1 NK:DBMSC ratios, several immune markers were evaluated by flow cytometry, and the expression recorded as mean fluorescent intensity (MFI). After 50 h of co-culture with DBMSCs, the expression of CD69 by NK cells was significantly decreased in all NK: DBMSC ratios, relative to untreated NK cells (Figure 6A, *P< 0.05), while the expression of NKG2D was significantly decreased at the 25:1 and 50:1 NK: DBMSC ratios (Figure 6B, *P< 0.05), as well as that of DNAM at a ratio of 100:1 NK: DBMSCs (Figure 6C, *P< 0.05). Finally, co-culturing NK cells with DBMSCs had no significant effect on their expression of NKp30, NKp44, NKp46 and CD94 (Figures 6D–G, P> 0.05).

**FIGURE 7:**
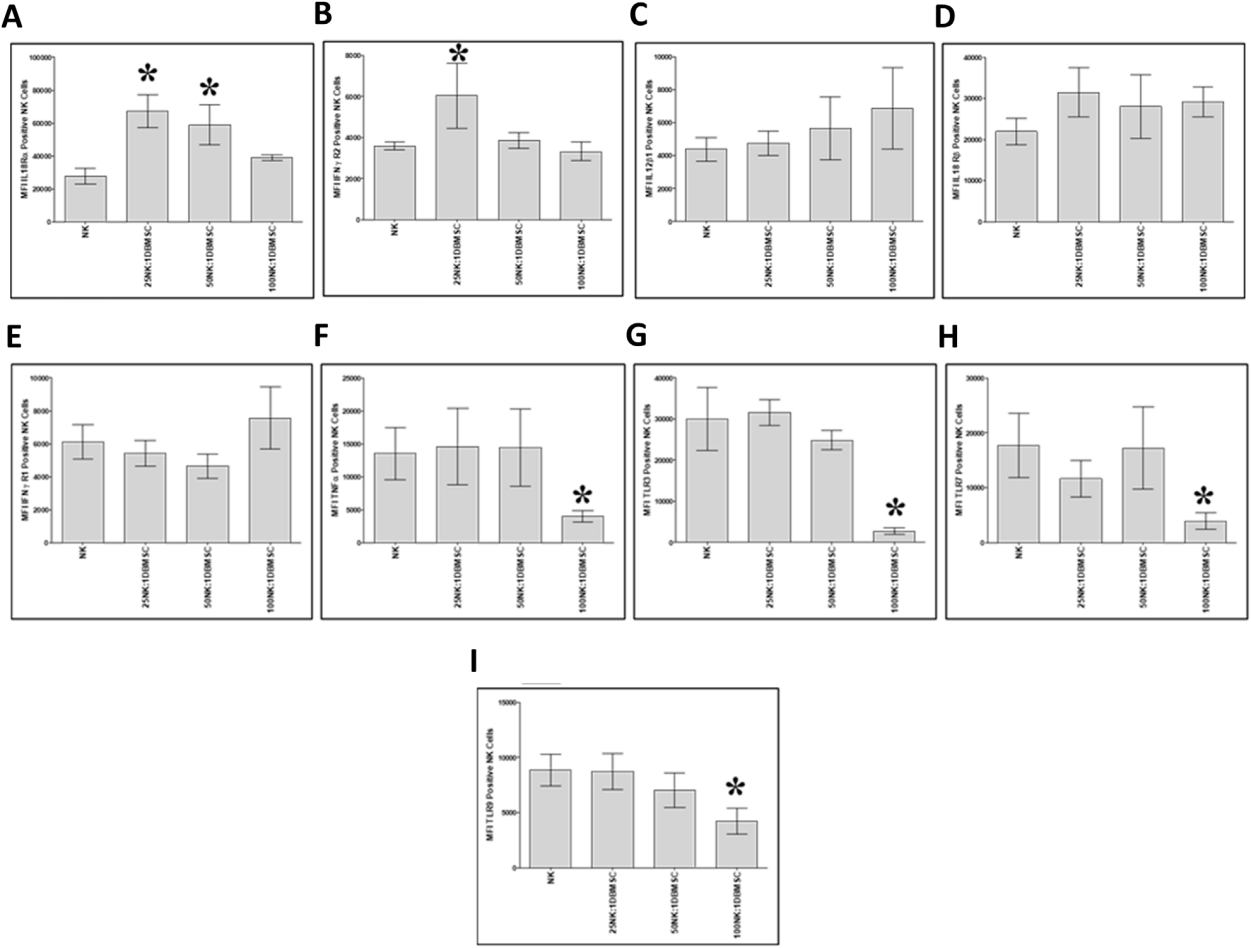
NK cell immune marker expression following co-culture with DBMSCs. Using flow cytometry, the expression of immune markers was evaluated in NK cells harvested from NK/DBMSC cytolytic experiments. Relative to untreated NK cells (NK), co-culturing NK cells with DBMSCs significantly increased NK cell expression of **(A)** IL-18Rα at the NK: DBMSC ratios of 25:1 and 50:1, and **(B)** IFN-ɤ R2 at the NK: DBMSC ratio of 25:1. Co-culturing NK cells with DBMSCs had no significant effect on NK cell expression of **(C)** IL-12Rβa, **(D)** IL-18 Rβ1 or **(E)** IFN-ɤ R105 at any of the indicated NK:DBMSC ratios, while it significantly decreased NK cell expression of **(F)** TNF-α, **(G)** TLR3, **(H)** TLR7, and **(I)** TLR9 at the NK: DBMSC ratio of 100:1. Experiments were performed in triplicate and repeated 10 times using using 10 independent NK/pMSC cytolytic experiments wherein the NK cells and pMSCs for each experiment were prepared from the peripheral blood of 10 healthy subjects and 10 normal human full-term placentae, respectively. Bars represent standard errors, *P< 0.05.

Next, we examined whether co-culturing NK cells with DBMSCs modulated their expression of the immune markers listed in Table 2. After 50 h of culture with DBMSCs, the expression of IL-18 Rα by NK cells significantly increased at the 25:1 and 50:1 NK:DBMSC ratios, relative to untreated NK cells (Figure 7A, *P< 0.05), while the expression of IFN-ɤ R2 significantly increased at the ratio of 25:1 NK:DBMSCs (Figure 7B, *P< 0.05). In contrast, the expression of TNF-α, TLR3, TLR7 and TLR9 was significantly decreased in NK cells at the 100:1 NK: DBMSC ratio (Figures 7F–I, P< 0.05). Co-culturing of NK cells with DBMSCs had no significant effect on their expression of IL-12Rβ1, IL-18 Rβ and IFN-ɤ R1 at all NK: DBMSC ratios (25:1, 50:1 and 100:1), relative to untreated NK cells (Figures 7C–E, P> 0.05).

## Discussion

In this study, we have investigated the interaction between DBMSCs and human NK cells. We found that exposure of resting non-activated NK cells (NK cells induced to proliferate by IL-2) to high numbers of DBMSCs reduced their proliferation (Figure 1A), while the exposure of IL-2-activated NK cells to high numbers of DBMSCs increased their cell proliferation potential (Figure 1B). The inhibitory effect of DBMSCs on resting non-activated NK cells is similar to that of MSCs from the chorionic villi of the fetal part of human term placenta (pMSCs) (Abumaree et al., 2019b), and MSCs from the bone marrow (BMMSCs) (Sotiropoulou et al., 2006; Spaggiari et al., 2006; Spaggiari et al., 2008). These effects were different for the MSCs isolated from the DPMSCs of human fetal membranes. The pro-proliferative effect of DBMSCs on IL-2-activated NK cells is similar to that of DPMSCs (Abumaree et al., 2018), and opposite to the inhibitory effect of pMSCs (Abumaree et al., 2019b) and BMMSCs on IL-2-activated NK cells (Sotiropoulou et al., 2006; Spaggiari et al., 2006; Spaggiari et al., 2008). One explanation for this is that the original microenvironment of these different MSC populations (i.e., their niche) might play a role in how MSCs influence the functions of NK cells. The mechanisms underlying the modulatory effects of DBMSCs on NK cell proliferation could be due to their secretion of specific soluble factors that have pro- and anti-proliferative functional activities. They include, IL-10, IL-12, and transforming growth factor beta-1 (TGF-beta 1) (Su et al., 1991; Naume et al., 1993; Cai et al., 1999); this possibility remains a question for future studies to address.

The cytolytic activity of NK cells against BMMSCs has previously shown to be stimulated by low expression of HLA class I molecules by the BMMSCs (Spaggiari et al., 2006). DBMSCs expressed significant levels of MHC class I molecules (Table 3), but were nevertheless lysed by NK cells (Figure 3), indicating that this cytolytic activity is independent of the expression of MHC class I molecules. This is similar to that of pMSCs and DPMSCs (Abumaree et al., 2018; 2019b). Our findings are in agreement with a previous report showing that expression of HLA class I molecules is not essential for NK cytolytic activity (Peña et al., 1990). We also confirmed this by showing that co-culturing NK cells with DBMSCs did not interfere their ability to lyse MCF-7 cells, which also express HLA class I antigens (Figure 5) similar to previously reporting for pMSCs and DPMSCs (Abumaree et al., 2018; 2019b).

Interestingly, preconditioning of NK cells by co-culturing them with high number of DBMSCs inhibited their cytolytic activity against MCF-7 cells (Figures 5C–E). This is opposite to the stimulatory effect of low numbers of DPMSCs and pMSCs on the cytolytic activity of NK cells against MCF-7 (Abumaree et al., 2018; 2019b). This may also be attributed to the microenvironment of these MSC populations. However, NK cells preconditioned by low numbers of DBMSCs had an anti-proliferative effect on MCF-7 cells (Figure 5H). These preconditioned NK cells expressed low levels of activating receptors, including CD69 and NKG2D (Figure 6), indicating that these receptors might mediate MCF-7 cells lysis by NK cells, as previously reported for pMSCs (Abumaree et al., 2019b) and DPMSCs (Abumaree et al., 2018). By contrast, the preconditioning of NK cells by co-culturing them with low numbers of DBMSCs did not inhibit their cytolytic activity against MCF-7 cells (Figure 5F). However, these preconditioned NK cells expressed low levels of activating receptors such as CD69, NKG2D, DNAM-1 and other molecules known to be involved in their cytolytic activity, including TNF-α, TLR3, TLR7 and TLR9 (Figures 7E,G–I) (Lauzon et al., 2006). Therefore, other activating receptors (e.g., NKp30, NKp44 and NKp46) or other mediators might mediate the lysis of MCF-7 cells by NK cells.

BMMSCs have been reported to produce a range of molecules with dual functions, such as IL-10, which stimulates NK cell cytolytic activity (Cai et al., 1999) and IL-6, which has anti-NK cell cytolytic activity (Cifaldi et al., 2015). Therefore, we believe that the experimental conditions or the cellular micro-environmental factors would determine the modulatory effects of DBMSCs on NK cells. Such a scenario could be investigated in future *in vitro* and *in vivo* studies.

DBMSCs expressed high levels of PVR ligand, which binds to DNAM, the NK cell activating receptor (Table 3). As expected, the blocking of DNAM by a monoclonal antibody inhibited the lysis of DBMSCs by NK cells. These results suggest the involvement of other NK cell activating receptors such as CD69, NKG2D, and NKp30 (Figure 4). In addition, DBMSCs also reduce NK cell expression of CD69 and NKG2D and, therefore, can directly inhibit the cytolytic activity of NK cells (Figure 6). In contrast, blocking of other NK cell activating receptors (NKp44 and NKp46) did not inhibit NK cell lysis of DBMSCs (Figure 4), indicating that these receptors as well as the NK cell inhibitory receptor CD94/NKG2A do not play a role in the cytolytic activity of DBMSCs. The role of NK cell activating receptors in NK cell cytolytic activity on DBMSCs is different to that of DPMSCs (Abumaree et al., 2018) and pMSCs (Abumaree et al., 2019a). This may also be linked to the microenvironment as indicated above.

As previously reported for DPMSCs and pMSCs (Abumaree et al., 2018; 2019b), only high number of IL-2-activated NK cells lysed DBMSCs (Figure 3) while resting, non-activated NK cells did not lyse the DBMSCs irrespective of their number. They however reduced their proliferation (Figures 2B–E). In a condition where IL-2-activated NK cells were low in number, the proliferation of DBMSCs was reduced (Figures 3G,H). DBMSCs were more resistant to lysis by NK cells than DPMSCs and pMSCs (Abumaree et al., 2018; Abumaree et al., 2019b). This resistance could be attributed to their niche in the placenta where DBMSCs are closer to the maternal vessels than DPMSCs and pMSCs, and are constantly exposed to harsh environment of inflammation and oxidative stress during normal human pregnancy (Cai et al., 1999; Spaggiari et al., 2008).

Lysis of DBMSCs by NK cells suggest that reparative activities of DBMSCs are mediated via paracrine mechanisms rather than cell-cell interactions, as was previously reported for DPMSCs and pMSCs (Abumaree et al., 2018; Abumaree et al., 2019b). DBMSCs produce molecules such as, IL-10, vascular endothelial growth factor (VEGF), TGFβ-1, and B7-H4 (Abomaray et al., 2016), which have immune and reparative modulatory properties. It is therefore highly likely that these secreted molecules mediate the modulatory effects of DBMSCs on the functions of immune cells and other cell types (Abumaree et al., 2013; Abumaree et al., 2017a; Abomaray et al., 2015). Future research is therefore necessary to reveal the mechanism underlying DBMSC functions on NK cells and other immune or non-immune cells.

NK cells preconditioned by a high number of DBMSCs increased their expression of IL-18 Rα and IFNγ R2 (Figures 7B,E). These data suggest that DBMSCs may enhance the anticancer activities of NK cells through their receptors IL-18 and IFNγ, which normally mediate the stimulatory effects of NK cells on cancer cells (Hafner et al., 1999; Wong et al., 2013; Mandai et al., 2016) as previously reported for DPMSCs and pMSCs (Abumaree et al., 2018; 2019b). This data indicate that DBMSCs might have a dual effect on NK cells, as shown here *in vitro*, where NK cells preconditioned with high number of DBMSCs inhibited NK cytolytic activity against DBMSCs, while having an anti-proliferative effect on MCF-7 cells. This may possibly be mediated through a mechanism that might involve, for example, the IL-18 receptor, which usually facilitates the secretion of molecules with anti-cancer activities, such as TNF-α and nitric oxide (Cheng et al., 2013).

Our data suggest that DBMSCs could be useful in treating cancers by enhancing the anticancer activities of NK cells. This may involve DBMSC-NK cell interaction after DBMSC transplantation in cancer patients, or *in vitro* by the NK cell adoptive transfer approach (Krause et al., 2004; Smyth et al., 2004; Tarek et al., 2012) where different mechanisms such as IL-18, IFN-ɤ receptors or other mediators may be involved. This therapeutic approach will be addressed in a future study, where we will identify the role of DBMSCs and the pathways involved in enhancing the anticancer activities of NK cells.

## Conclusions

Our data suggest that DBMSCs have the potential to modulate the functions of NK cells toward cancer cells, and therefore, are promising therapeutic agents for cancers.

## In Memoriam

This paper is dedicated to late “Professor Mohammad Abumaree”, who was the main architect of this study. We will continue to be inspired by his dedication and profound passion for Stem Cell research at KAIMRC. May his soul rest in peace.

## Data Availability

The original contributions presented in the study are included in the article/supplementary material, further inquiries can be directed to the corresponding author.
